# Emerging Concepts for Myocardial Late Gadolinium Enhancement MRI

**DOI:** 10.2174/1573403X113099990030

**Published:** 2013-08

**Authors:** Adelina Doltra, Brage Høyem Amundsen, Rolf Gebker, Eckart Fleck, Sebastian Kelle

**Affiliations:** 1 Thorax Institute, Hospital Clínic, Institut d'Investigacions Biomèdiques August Pi i Sunyer, University of Barcelona, Spain;; 2 MI Lab and Department of Circulation and Medical Imaging, Norwegian University of Science and Technology, MTFS, Trondheim, Norway;; 3 Department of Internal Medicine/Cardiology, Deutsches Herzzentrum Berlin, Germany

**Keywords:** Cardiac, late gadolinium enhancement, magnetic resonance imaging, CMR, viability.

## Abstract

Late gadolinium enhancement is a useful tool for scar detection, based on differences in the volume of distribution of gadolinium, an extracellular agent. The presence of fibrosis in the myocardium amenable to be detected with late gadolinium enhancement MRI is found not only in ischemic cardiomyopathy, in which it offers information regarding viability and prognosis, but also in a wide variety of non-ischemic cardiomyopathies. In the following review we will discuss the methodological aspects of gadolinium-based imaging, as well as its applications and anticipated future developments.

## BACKGROUND

### Myocardial Viability

Viability means living, and myocardial viability refers to myocardium that is living, and contributes to, or has the potential to contribute to, systolic ejection of blood. The term is mostly used in relation to decisions on revascularization in patients with coronary artery disease. In patients with reduced left ventricular function it is important to determine whether myocardial tissue with reduced function and reduced blood supply will actually benefit from re-establishing blood supply. In this setting, the gold standard for viability is a retrospective one: the ability to improve function after revascularization. Accurate viability tests are important for diagnostic and prognostic purposes and, although retrospective studies suggest large benefits, there is a striking lack of prospective randomized controlled clinical trials determining the effect of viability testing on morbidity and mortality in patients with coronary artery disease.

Before the introduction of gadolinium contrast agents, there were two main principles for viability testing: function and metabolism. The first is utilized in low-dose dobutamine stress, where an improvement in function from rest to 10 (-20) microgram/kg/min of dobutamine is used as a marker of viability. This can be done with either echocardiography or magnetic resonance imaging [1]. The second principle, metabolism, is exemplified by positron emission tomography, which can demonstrate uptake of radioactively labelled glucose and simultaneously quantify blood flow in the same tissue [2, 3].

With the introduction of gadolinium contrast agents in cardiac MR, a new principle was established: late gadolinium enhancement imaging (LGE). The concept is based on the delayed wash in and wash out in tissue with an increased proportion of extracellular space. In the setting of an acute myocardial infarction, this is caused by cellular necrosis and lysis, and edema, while in the chronic infarcted tissue, the fibrous scar tissue with its increased extracellular space is the basis. The increased amount of gadolinium can be demonstrated by T1-weighted imaging, in the time period of 10-30 min after contrast administration.

While late gadolinium enhancement was originally developed to image scar, it has also been found useful in other kinds of heart disease, mainly in the diagnosis of cardiomyopathies. Different kinds of cardiomyopathies exhibit different patterns of LGE, which opens up entirely new possibilities in the differential diagnosis in patients with reduced ventricular function. LGE is based on differences in extracellular space in different areas of myocardium and is therefore only useful if the disease is regional (e.g. myocardial infarction scar, sarcoidosis). In diseases that affect the whole myocardium the method runs into trouble. The most typical example is amyloidosis but general fibrosis also seems to be an important part, or common pathway, of the unfavorable remodelling and hypertrophy seen in, for instance, heart failure, aortic stenosis and hypertensive heart disease. Fibrosis is also a part of normal aging [4]. 

In the following we will discuss the methodological aspects of gadolinium-based imaging, and also its applications and possible future developments.

## METHODOLOGY

### Gadolinium Contrast Agents

Gadolinium (Gd) is an element and is toxic in its unbound state. Therefore, the contrast agents (CA) consist of a large molecule with the sole task of keeping Gd bound during intravenous injection. There are three different groups of carrier molecules with different affinity to Gd (cyclic, non-cyclic and others), with the cyclic carriers considered safest (i.e. least likely to release Gd). The carrier molecules are relatively large and do not enter the intracellular space. Thus they are classified as extracellular contrast agents. The ones with the largest molecular size only extravasate to a limited degree and can be said to be vascular agents, like the gadolinium-based albumin-bound CA [5]. An ideal chelating agent has to be stable (in order to avoid liberation of unbound Gd), achieve enough increase of signal intensity and be soluble in water (to be easily administered to the patient). 

Most of the CA are excreted by the kidneys, having a half-life of 2 hours and being almost completely cleared from the bloodstream after 24 hours. Although it has been considered a safe drug for years, we now know that Gd can cause a rare, albeit severely debilitating disease known as nephrogenic systemic sclerosis (NSF). This condition, which resembles scleroderma, is mainly seen in patients with reduced kidney function, especially those who are in dialysis and have undergone multiple angiographic exams with high doses of Gd. After the establishment of rigorous guidelines for the use of Gd-CA in patients with reduced renal function, there have been very few new cases. Some of the CA thought to have the lowest affinity to Gd have also largely been abandoned. This potential toxicity of current Gd CA has prompted the search for new MRI CA without this limitation. 

### MR Imaging for LGE – technical Issues

Gd is a paramagnetic element and reduces T1 time. Thus, T1 weighted sequences like fast low-angle shot (FLASH) can be used. The contrast to noise ratio was dramatically improved with the introduction of the inversion recovery principle (IR), which effectively nulls the signal from normal myocardium. 

The technique used for LGE consists of an injection of a 0.1-0.2 mmol/kg bolus of Gd-based contrast, and after 10-20 minutes the start of acquisition of T1-weighted images with an adequate inversion time (TI) to null the signal from normal myocardium. Choosing a correct TI is of crucial importance to obtain images with enough diagnostic quality. Optimal TI varies in each patient and in accordance with certain factors such as cardiac output or contrast dosage. The optimal TI is that which results in suppression of normal myocardium (with low signal and, thus, dark image) along with a brighter signal from the myocardial cavity and a very bright signal in the scar tissue. An optimal TI should enable the examiner to differentiate endo-, mid- and epicardial LGE, and also to see possible intracavitary thrombi.

The TI can be determined either by trial and error, which is effective for experienced examiners, or by using a Look-Locker sequence, that acquires a series of images with variable TI during one breath-hold, and lets the examiner pick the one with the most favorable contrast. It has also to be taken into account that the TI must be increased during the examination if LGE images are acquired over a period of more than 5 min. 

In order to overcome, at least partially, the disadvantages of TI selection, phase-sensitive inversion recovery (PSIR) techniques have been developed. Those techniques, available on some commercial scanners, permit the acquisition of DE images without having to pick a precise TI [6]. 

### Physiopathologic Principles of Gadolinium Imaging

Gadolinium contrast medium has a molecular size that allows it to distribute in the extracellular space without entering the myocardial cells in normal conditions. However, under certain pathologic circumstances, either the extracellular space may increase or the myocardial cell membrane may be disrupted, leading to an increase in the volume of distribution of gadolinium and, as a consequence, gadolinium enhancement. In acute myocardial infarction, for instance, cellular necrosis is associated with cell membrane rupture and increase in gadolinium distribution [7]. On the other hand, in a scar secondary to a chronic myocardial infarction the extracellular space will be increased due to collagen deposits [8]. As will be discussed later on, non-ischemic cardiomyopathies may also exhibit LGE, due to either of the two mechanisms described above. 

### Assessment of LGE

The threshold that should be used to define hyperenhancement is not clear. In clinical practice, LGE is evaluated qualitatively by visual estimation, establishing a percentage of the thickness of infarcted myocardium in relation to the global wall, in order to define the transmural extent of LGE. A semiquantitative approach has also been described, using the American Heart Association 17 segment model and giving each segment a score from 0 to 4 depending on the extent of scar (0: no scar, 4: 100% scar). A final score is given by the summation of all segments’ scores and division of the result by 17. 

In addition to visual estimation, some commercially available softwares for quantification of scar have also been developed. However, the exact threshold to define scar is still not clear. Kim*, et al.* [9] used 2 SD above normal remote myocardium, although some concern exists on the possibility of overestimating scar size using this approach.

### Correlation to Histopathology

Hyperenhancement on LGE has a close correlation to histopathology proven myocardial necrosis, as demonstrated by some studies. In an experimental study, Kim and colleagues [9] demonstrated with ex-vivo MRI that the extent of hyperenhancement was the same as the spatial extent of myocardial necrosis at 1 and 3 days after infarction, and the scar at 8 weeks. In another experimental study, semi-automatic techniques for analysis of scar demonstrated the best correlation with post-mortem evaluation [10]. Also, the reproducibility of LGE for infarct size determination has also been demonstrated [11].

### Limitations of LGE T1 Mapping

In addition to the variability of TI between different subjects and conditions, a second drawback of LGE is that the scar size will be somewhat different depending on the time delay after contrast injection. This disadvantage, however, is probably less important in a clinical setting than in research, where quantification is essential.

Perhaps the main drawback of the LGE technique is its limitation in evaluating diffuse fibrosis. Since the enhanced area is defined on the basis of the difference in signal intensity relative to that of the normal myocardium, if there is diffuse fibrosis no differences in signal intensity will be observed. In order to overcome this limitation, T1 mapping techniques have been developed that allow the quantification of the relaxation times of the myocardium, with or without administration of Gd agents. The most frequently used sequence is the modified Look Locker inversion recovery (MOLLI) sequence [12]. Although promising, however, to date there are few studies that have applied T1 mapping in a clinical setting. 

## CLINICAL APPLICATIONS OF LGE

### Acute Ischemic Heart Disease

Currently LGE MRI is recommended by the scientific societies for scar detection [13] andis a very accurate technique for the detection of acute myocardial infarction (MI), even in cases with a discrete elevation of necrosis enzymes and no ECG changes or wall motion abnormalities [14]. Even more importantly, due to its higher spatial resolution (1.4 x 1.9 x 6 mm for MRI in comparison to approximately 10 mm in-plane for SPECT [15]) LGE-MRI detects infarcts better than SPECT, especially subendocardial infarctions that are missed in SPECT (47% missed subendocardial infarcts in one study [15]). MRI can also detect the ischemic area at risk after a coronary occlusion. This area, which shows high intensity in T2-weighted black blood images, will not show enhancement on LGE MRI, helping to differentiate it from the infarcted zone. 

LGE-MRI can also offer some important prognostic data. The extent of enhanced myocardium correlates with biomarkers of myocardial necrosis and left ventricle (LV) ejection fraction (EF) at late follow-up in patients with acute MI, as demonstrated in one work [16]. Also, in patients with reperfused acute MI, the transmural extent of infarction predicts improvement in contractile function and LV remodelling. No differences in functional improvement at follow-up were demonstrated in one study between no enhancement and enhancement of up to 25%, but significant differences were found with enhancement superior to 25% of wall thickness [17]. In another work, improvement in contractility was not predicted by enzyme levels or total infarct size but by the extent of dysfunctional myocardium without enhancement or with enhancement <25% [18].

LGE MRI is also a useful tool to detect microvascular obstruction (MVO). The visualization of MVO in LGE MRI is that of a subendocardial area of low signal intensity surrounded by enhancement. The detection of MVO has prognostic implications as well, since it has demonstrated correlations with adverse events (heart failure, arrhythmia and even death), bigger infarct size, lower EF and LV remodelling [19, 20].

Finally, in an acute MI setting, LGE MRI can be useful to detect the extent of the infarct peripheral zone. This peripheral zone, which possesses lower signal intensity than that of the infarct core, corresponds to an area with both viable and non-viable myocytes. A larger peripheral zone has been related to an adverse prognosis with higher mortality at follow-up due to arrhythmia [21].

### Chronic Ischemic Heart Disease

As has already been discussed, both acute and chronic scars from MI show LGE. In order to differentiate between the two, the presence of wall thinning as well as T2-weighted imaging may be useful; whereas wall thinning suggests chronic infarction, high-intensity on T2 is due to the edema of an acute MI.

**Fig. (1):**
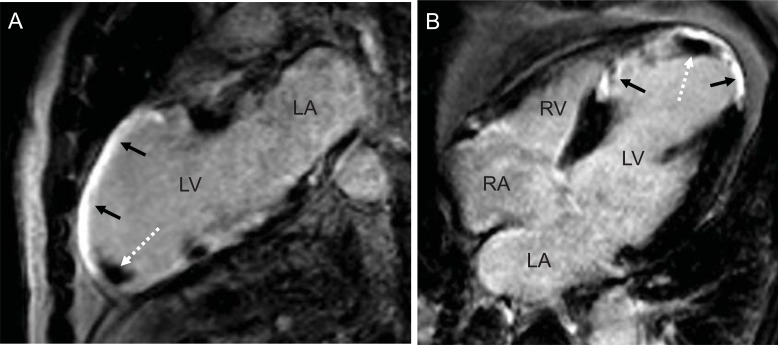
Two - (**A**) and four-chamber-view (**B**) of a patient with coronary artery disease and history of a large anterior and septal myocardial infarction (black arrows) with development of a small thrombus in the apex (white dotted arrow). (LV: left ventricle, LA: left atrium, RA: right atrium, RV: right ventricle).

In chronic ischemic heart disease (IHD), the major contribution of DE MRI is to predict recovery of function after revascularization. The presence and extent of LGE are strongly associated with the probability of improvement in contractility (Fig. **1**). Kim and colleagues [22] studied patients with LV dysfunction scheduled for surgical or percutaneous revascularization, finding a progressive decrease in the likelihood of improvement after revascularization as the transmural extent of hyperenhancement increased (contractility increased in 78% of segments without enhancement, but only in 1 of 58 segments with hyperenhancement >75% of wall thickness). Further studies have also confirmed these findings [23].

Finally, another application of LGE in chronic IHD is to differentiate between ischemic etiology and non-ischemic etiology in dilated cardiomyopathy [24]. In contrast to non-ischemic cardiomyopathy, IHD shows enhancement that is subendocardial or transmural and affects an area concordant with a coronary artery supply territory.

### LGE MRI in Non-ischemic Heart Disease

Several cardiomyopathies may present with different patterns of LGE, but these patterns will either not affect the subendocardium or not correspond to any coronary territory. LGE in non-ischemic HD may affect the mid-wall or the epicardium, with either a diffuse or focal distribution.

### Myocarditis

Cardiac magnetic resonance (CMR) imaging has emerged as an effective tool for the diagnosis of myocarditis, with high values of specificity [25]. The pattern of late gadolinium enhancement in patients with myocarditis can be intramural (mainly of septal localization) or, more frequently, subepicardial with a patchy distribution (normally localized in the basal and mid-ventricular segments of the posterolateral wall) [25, 26]. The presence of late gadolinium enhancement has also been associated with biopsy-proven inflammation in the same area [26]. 

CMR has a good diagnostic performance (80%) in patients with chest pain, positive necrosis enzymes and absence of coronary artery disease, but its diagnostic capability improves when associated with endomyocardial biopsy (reaching 95%). There is a good diagnostic agreement between CMR and endomyocardial biopsy for the diagnosis of myocarditis (kappa=0.7) [25]. According to one study [27], the accuracy of LGE for the diagnosis of acute myocarditis was 71%, which increased to 85% when at least one other CMR sequence was also positive for myocarditis (either T2-weighted imaging or T1-weighted imaging early after gadolinium administration).

Apart from its diagnostic utility, the presence of LGE in patients with myocarditis seems also to have prognostic implications [28]. In a recent study, LGE was found to be the best independent predictor of overall and cardiovascular mortality in a population with biopsy-proven myocarditis, with hazard ratio superior to that of LV ejection fraction, LV end-diastolic volume or NYHA functional class [29].

Finally, new emerging CMR techniques may also provide some new insights. The usefulness of CMR with magneto-fluorescent nanoparticles (MNP) for detecting myocarditis has been evaluated in a recent experimental study, in which it was demonstrated that MNP CMR allowed better visualization of myocardial inflammation than conventional CMR [30].

### Hypertrophic Cardiomyopathy

**Fig. (2):**
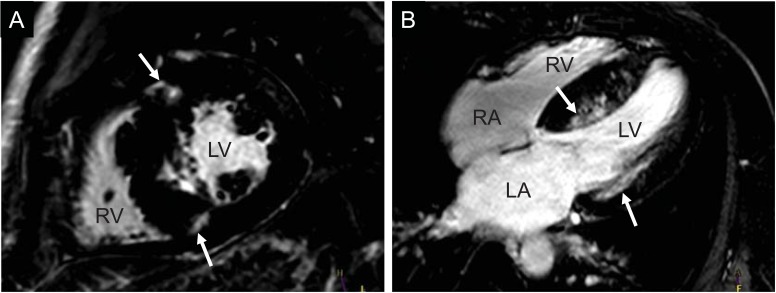
Presence of localized hyperenhancement (white arrows) in a patient with hypertrophic cardiomyopathy at the junction points of the right ventricle in the septum (A, basal short axis orientation), as well as in the subepicardial lateral and septal mid-wall in the LV (B, 4-chamber-view). (RV: right ventricle, LV: left ventricle, RA: right atrium, LA: left atrium).

The presence of hyperenhancement in hypertrophic cardiomyopathy (HCM) is usually localized in the junction points of the right ventricle (RV) in the septum (Fig. **2**), as well as in the subepicardial and mid-wall in the LV [31]. The importance of studying LGE in HCM is due to the fact that the disease has been associated with risk of malignant arrhythmias and sudden death. In a population with HCM without significant clinical symptoms, the presence of LGE was associated with a greater likelihood of ventricular arrhythmias in 24h Holter monitoring [32]. In another work, the presence of scar in a population of asymptomatic patients with HCM was an independent predictor of all-cause and cardiovascular mortality [33].

### Amyloidosis

The typical characteristic of this condition on MRI is the difficulty of choosing an optimal TI value to null the myocardium, due to the deposit of amyloid. LGE is found in most patients (76%) with cardiac amyloidosis according to one study [34]. The pattern of LGE can be variable, with both subendocardial and subepicardial localizations and patchy or diffuse distributions.

### Idiopathic Dilated Cardiomyopathy

Most patients will show no enhancement, whereas others may present with mid-wall hyperenhancement [24]. In this study by McCrohon*, et al.*, 13% of the patients with dilated cardiomyopathy and without significant coronary disease in the angiography showed a LGE pattern indistinguishable from that of the patients with IHD, suggesting a coronary recanalization after MI.

### Other Non-ischemic Conditions

In sarcoidosis the LGE pattern is usually midventricular or subepicardial, and affects specially the basal segments. Right ventricular cardiomyopathy may present with LGE affecting either the RV free wall or the septum. Chagas disease may show a pattern of LGE that is either transmural, subepicardial or subendocardial, with the inferolateral segments being the most frequently affected. Finally, the association of biventricular subendocardial enhancement with adjacent thrombus is typical for endomyocardial fibrosis.

### Cardiac Resynchronization Therapy

Several studies have assessed the role of DE-CMR in cardiac resynchronization therapy (CRT), showing an association between the presence of myocardial scar and lack of response to this therapy. It was demonstrated in one study [35] that a percent total scar of ≤15% accurately identified patients with clinical response to CRT. In another study with 40 patients, Bleeker and colleagues [36] found a low clinical response rate to CRT in patients with posterolateral scar (14% vs 81% in patients without posterolateral scar), independently of the presence of baseline LV dyssynchrony. Finally, the negative impact of pacing over scar was further demonstrated in another work [37], in which scar transmurality ≥51%, scar size ≥33% and pacing over a posterolateral scar were related with a suboptimal clinical response to CRT. 

However, given the relative contraindication of performing a CMR in the presence of a CRT device, the use of CMR in this subset of patients is limited to the pre-implant evaluation, using other imaging techniques for the patient follow-up.

### Atrial Fibrillation

Recently, DE-MRI has also proved its utility in the field of atrial fibrillation (AF). Specifically, the group of Utah has applied this technique in the left atrium, allowing for the visualization of DE in this particular cardiac chamber [38]. In a work with 81 patients with AF undergoing catheter ablation [39], the extent of DE in the left atrium was able to predict the success of this procedure: while 75% of the patients with extensive left atrium enhancement presented AF recurrence after ablation, it was only present in 14% of the patients with minimal enhancement and 43.3% of patients with moderate DE. The association between left atrium DE and stroke was also investigated in another study by the same group [40], in which patients with previous stroke had a significantly higher percentage of left atrium DE compared to patients without stroke history.

### Detection of Inflammation: ^19^F MRI

In order to detect inflammatory areas, contrast agents that infiltrate inmunocompetent cells have been used, allowing these cells to be tracked and, as a consequence, effectively delimiting inflammatory regions. Two possible approaches are used: one, using paramagnetic nanoparticles, and the second, using fluorine isotope. The latter has the advantage of lack of background signal and, thus, is highly specific. 

Perfluorocarbons (PFCs) which contain a high concentration of ^19^F atoms are used. PFCs are taken up by phagocytic cells and accumulate in inflammatory regions, offering the possibility of detecting inflammation and events to monitor treatment. However, it is still unresolved how to widely apply this technique in clinical routine [41].

## CONCLUSION

LGE MRI is a valuable tool for scar detection, as recommended by the principal scientific societies, based on the differences in volume distribution of gadolinium between normal tissue and that of fibrosis or necrosis. In ischemic cardiomyopathy its role in guiding revascularization is essential and it also offers prognostic information. Other myocardial diseases may also show different patterns of LGE, pointing out the usefulness of this technique in the diagnosis and even prognosis of some of these entities. Finally, new techniques are evolving that may generate novel applications of this technique in the future.
